# Effect of Styrene Polymerization on the Bondability of Beech and Alder Wood with Different Adhesives

**DOI:** 10.3390/ma17246212

**Published:** 2024-12-19

**Authors:** Emil Żmuda, Anita Wronka, Grzegorz Kowaluk, Andrzej Radomski

**Affiliations:** 1 Department of Wood Science and Wood Protection, Institute of Wood Sciences and Furniture, Warsaw University of Life Sciences—SGGW, Nowoursynowska Str. 159, 02-776 Warsaw, Poland; emil_zmuda@sggw.edu.pl (E.Ż.); andrzej_radomski@sggw.edu.pl (A.R.); 2 Department of Technology and Entrepreneurship in the Wood Industry, Institute of Wood Sciences and Furniture, Warsaw University of Life Sciences—SGGW, Nowoursynowska Str. 159, 02-776 Warsaw, Poland

**Keywords:** polymerization, alder, beech, styrene, wood, bondability, wood bonding, shear strength

## Abstract

This study aimed to evaluate the bondability of beech and alder wood modified through styrene polymerization within the wood lumen. Unmodified wood samples served as the reference material. Bondability was tested using four adhesive types commonly used in wood technology: polyvinyl acetate (PVAc), urea-formaldehyde (UF), phenol-resorcinol-formaldehyde (PRF), and epoxy resin. In addition to shear strength measurements, the adhesive density profile was also assessed. Results indicated that styrene modification generally reduced wood bondability, with reductions in shear strength ranging from 8% to 23% for beech wood and 1.6% to 29% for alder wood, depending on the adhesive type. The only exception was observed with the epoxy adhesive, which showed a 13% improvement in bonding quality for modified wood. These findings suggest that while styrene modification may enhance specific properties of wood, it can adversely affect its adhesion performance with some adhesive systems, except epoxy, which displayed improved compatibility with styrene-modified wood. The study offers insights for selecting suitable adhesives when using modified wood in structural applications.

## 1. Introduction

Wood modification alters its chemical, physical, and mechanical properties to enhance durability, increase resistance to external factors, and improve the material’s overall functionality [1,2]. Despite its many advantages, such as renewability and esthetic appeal, natural wood has certain limitations, including low resistance to moisture, susceptibility to fungi and insects, and limited dimensional stability [3,4]. For years, extensive research has focused on developing various methods to modify wood to enhance its properties and make it a more resistant and durable building material. This involves modifying key wood components such as cellulose, hemicelluloses, and lignin [1]. The most common chemical wood modification methods include furfurylation, acetylation, maleoylation, succinylation, modification with N-methylol acryl amide, reactive linseed oil derivative, methylated melamine resin, and citric acid [5,6,7]. Physical methods enhance wood surface modification through thermal treatment, impregnation, and cold plasma treatment. Thermal modification alters the properties of wood by applying heat, pressure, and moisture without additional chemicals [8,9]. We also distinguish wood surface modification, which involves treatments that alter only the outermost layers of the wood, explicitly targeting improvements in surface properties such as enhanced adhesion, increased weather resistance, reduced hygroscopicity and surface activation, and increased resistance to decay [8,10]. However, it should be pointed out that the structure of bonded lignocellulosic material, like bamboo, can be challenging and provide interesting results [11]. Even the combination of bonded wood species, like beech and alder, bonded with PRF, can improve bending features in homogenous beams [12].

The chemical modification of wood using styrene involves impregnating the wood with styrene and a cross-linking monomer, resulting in enhanced dimensional stability and improved mechanical properties [13]. Polymerizing wood, primarily through impregnation with liquid vinyl monomers followed by in situ polymerization, mainly deposits the polymer within the wood’s natural cavities. This process fills voids and enhances the wood’s resistance to dents and wear [14]. Impregnating wood with monomers or other agents followed by polymerization alters its chemical composition, significantly improving its properties. However, this process also increases the wood’s weight as the impregnating agents fill its cavities, which may limit its suitability for specific engineering applications [15]. The study demonstrates that lignin removal through delignification, followed by polymerization, enhances hydrogen bond formation between adjacent fibers during pressing. This process reduces the compressed product’s set recovery and increases the compacted samples’ specific tensile strength [16]. The polymerization of wood within its lumens using monomers such as styrene, butyl methacrylate, or methacrylates significantly improves water resistance and dimensional stability. Studies on black poplar (*Populus nigra* L.) showed an 85% reduction in water absorbability and a 50% decrease in volume swelling due to modification with styrene and maleic anhydride [17,18].

In the case of wood modification with styrene, as demonstrated using pine, there was a noticeable increase in thermal stability and a significant improvement in mechanical properties [19]. The impregnation of pine wood with styrene and glycidyl methacrylate (GMA) significantly enhanced its physical properties, including reduced water absorption, lower swelling, and increased hardness, indicating stronger interactions between the wood and the polymers [20,21]. Adding GMA to styrene resulted in substantial improvements in the modulus of rupture, elasticity, compressive strength, and hardness of wood–polymer composites, effectively overcoming the bonding challenges associated with wood after styrene polymerization [22]. Life cycle assessment (LCA) studies reveal that styrene-modified wood-plastic composites (WPCs) are more eco-friendly compared to unmodified versions, especially when they contain equal or greater amounts of wood fiber [23]. Modifying wood with maleic anhydride led to the enhanced modulus of rupture, modulus of elasticity, internal bond strength, and a slight reduction in thickness swelling. Wood modified with maleic anhydride shows promising potential for medium-density fiberboard (MDF) applications [24]. Modifying wood with maleic anhydride and similar compounds offers enhanced durability and increased resistance to decay-causing fungi, thereby reducing the reliance on environmentally harmful chemicals [25,26].

Research highlights the challenges of bonding wood after polymerization. While sanding is an obvious solution—sandblasting, sanding, or blasting enhances surface roughness and increases the contact area between the substrate and adhesive, thereby optimizing adhesion [27,28], and studies suggest that pre-activating the surface with hydrogen peroxide solutions significantly improves the shear strength of lap joints when using epoxy adhesives. This approach offers a promising solution to adhesion issues between wood and styrene polymers post-polymerization [29]. Furthermore, studies suggest that wood modified in this manner responds well to specialized adhesive mixtures, such as the SP adhesive, which comprises styrene-butadiene rubber (SBR) and polyethylene glycol (PEG). Additionally, ozonation has been found further to enhance the wood’s strength parameters [30].

Utilizing styrene-polymerized wood in construction projects can enhance the use of lower-value wood instead of high-quality timber, leading to significant cost savings.

Styrene-polymerized wood exhibits superior dimensional stability, rot resistance, and weathering durability, making it well-suited for various applications, including construction, infrastructure, and interior decoration [31]. The study revealed that chemical and thermochemical modifications to European beech wood significantly enhanced its suitability for indoor applications, such as kitchen tools [32]. Additionally, the properties of wood coatings derived from styrene-grafted natural rubber latex were evaluated, highlighting the potential of eco-friendly, water-based coatings for wood protection [33]. Incorporating styrene-polymerized wood into wood–polymer composites can enhance the structural performance of fast-growing, low-density woods, boosting their competitiveness in manufacturing processes [34] and using styrene-polymerized wood in construction projects can result in substantial cost savings compared to employing high-quality lumber [35,36].

The wood modification process increases the lifespan of wood products, prolonging the amount of atmospheric carbon sequestered inside the harvested wood products (HWP) material pool. Additionally, this lengthens maintenance cycles, positively affecting the environment and the economy [37].

Despite promising results in enhancing wood properties, the polymerization of styrene within the lumen of wood vessels remains primarily in the research and development phase. Although the method is not yet widely adopted in commercial applications, it holds significant potential for future use across various industries, provided safety and environmental impact concerns are adequately addressed [38]. Research suggests that these composites offer considerable promise for applications in sectors such as outdoor furniture—due to their resistance to moisture and UV radiation, they can be used for decking boards, fences, or elements of small architecture [39,40]—manufacturing and construction materials, such as load-bearing elements, like beams, columns, or planks [41]. However, considerable emphasis is still placed on advancing technologies that will optimize the performance and stability of these composites [42,43,44].

As described in various patents, efforts have been made to improve the polymerization process. Some of these patents highlight the use of additives that can enhance the impregnation process and improve the properties of the resulting composites. Patent US20200023546A1 discusses using additives such as organic catalysts (e.g., peroxides) in the styrene polymerization process, which helps improve curing efficiency. Additionally, it mentions using inhibitors like pinene to control the polymerization reaction, addressing some of the issues associated with wood polymerization [45]. Patent US3663261A describes a method for preparing plastic-impregnated wood using radiation. This patent demonstrates the development of wood polymer impregnation technologies, including styrene, through gamma radiation. It represents a process still in development, as it involves radiation-based and chemical processes that are being investigated but are not yet widely used in industry [46]. Patent WO1995017438A1 refers to applying various polymers, including styrene, in wood impregnation processes with additives that improve the properties of the resulting wood–polymer composites [47]. Patent WO2001053050A2 presents a method for wood impregnation, where wood is treated with organic monomers that polymerize within its structure, forming a wood–polymer composite. This process aims to enhance the mechanical properties of wood, such as moisture resistance, compressive strength, hardness, and dimensional stability, by introducing a polymer that penetrates the wood’s microstructure. A key aspect of this method is using organic monomers that can polymerize directly within the wood, allowing for the desired properties without the need for complex composite manufacturing techniques. Examples of monomers used in this process include styrene, acrylate, and methacrylate, which, once impregnated into the wood and polymerized, become an integral part of the material, improving its durability, weather resistance, and mechanical properties [48].

The latest patents related to styrene polymerization in wood are primarily in the developmental stage, with promising applications in enhancing wood properties such as dimensional stability and resistance to moisture. For instance, WO2024204320 (filed in 2024) discusses an innovative method to improve the mechanical properties of wood by incorporating styrene and other additives during the polymerization process, resulting in enhanced durability and structural integrity [49]. Similarly, WO2024008911 explores a process to modify wood’s hydrophobicity and dimensional stability by incorporating styrene monomers, offering increased resistance to environmental factors like humidity and temperature fluctuations [50]. Based on a review of patents, it can be observed that the research in this field has a long history but remains highly relevant today, with significant potential for broader application.

This study investigates the effect of styrene polymerization in wood, using 2% maleic anhydride and 1% benzoyl peroxide, on shear strength when impregnated wood was bonded with four different wood adhesives.

## 2. Materials and Methods

### 2.1. Materials

In a study that required lumen wood modification, two wood species were used: beech (*Fagus sylvatica* L.) and alder (*A. glutinosa* (L.) Gaertn). The moisture content of the wood used was 8.49% ± 0.24% (beech) and 8.37% ± 0.26% (alder), determined by the dry weight method. All the samples were conditioned in 20 °C ± 1 °C/65% ± 2% relative humidity for 7 days. For modification, samples were selected randomly for each series of 30 samples.

The styrene (MY-CHEM GmbH, Innungsstraße 11, 21244 Buchholz i.d. Nordheide, Germany) with 2% maleic anhydride (Thermo Scientific, 5781 Van Allen Way Carlsbad, CA, USA, manufactured in Belgium) and the initiator, 1% benzoyl peroxide (Sigma-Aldrich, Merck KGaA, Darmstadt, Germany, EMD Millipore Drive Corporation 400 Summit Drive, Burlington, MA, USA, manufactured in Germany), were used in this research. This study employed four types of adhesives: PVAc (Titebond Iii Ultimate Wood Glue, Franklin International, 2020 Bruck Street, Columbus, OH, USA), PRF and Epoxy (were supplied by the Research and Development Centre for Wood-Based Panels Sp. z o.o., Czarna Woda, Poland), and UF (Silekol Sp. z o.o., Kędzierzyn-Koźle, Poland).

### 2.2. Methods

The wood samples were cut to dimensions of 150 × 20 × 5 mm and then modified. The in situ modification process involves selecting the samples, weighing them, and measuring them. The samples are then flooded with a mixture of monomers, such as styrene with 2% maleic anhydride and the initiator, 1% benzoyl peroxide. They were loaded in a way that the mixture would not flow out, and spaces were left between them. The process used in this case involved pumping out air to below 100 mbar, and a vacuum was maintained by repeating the process every 30 min. Then, atmospheric-level pressure was allowed for 30 min into the chamber, as delivered by VacuumChambers.eu (Jodłowa St. 3A/34, 16-001 Ignatki-Osiedle, Poland). After this process, a vacuum was applied for 7 h 30 min, and then atmospheric pressure was again allowed for 30 min. The samples were removed from the solution, and the surfaces were wiped of excess monomer and placed in both films. The films were then sealed using a heat sealer. After the previously included preparation, the samples were placed in a dryer heated to 80 °C for 24 h, after which the temperature was changed to 105 °C for another 24 h. The next step was to release the samples from the foil and loosely arrange them in the dryer for a minimum of 24 h at 105 °C. After cooling in the desiccator, the samples were measured and weighed to determine the density and weight percent gain (WPG) parameters. The WPG was calculated according to the method of Venas and Rinnan [51].

For wood bonding, the adhesive amount applied to each sample was adjusted to ensure the dry matter content was consistent across all tested samples. The reference spread was 200 g m^−2^ of UF binder. The polymerized samples were sanded with 120-grit sandpaper before gluing to remove the exceeding polymer layer. All the bonded samples were conditioned in 20 °C ± 1 °C/65% ± 2% relative humidity for 7 days. All tests presented in the article complied with the applicable European standards: adhesive dry matter content was determined according to EN 827 [52] and shear strength according to EN 205 [53] on a computer-controlled universal testing machine (Research and Development Centre for Wood-Based Panels Sp. z o.o., Czarna Woda, Poland). The density profile was analyzed with a GreCon DA-X device (Fagus-GreCon Greten GmbH & Co. KG, Alfeld/Hannover, Germany) through direct X-ray densitometry, measuring the panel thickness in 0.02 mm steps. A minimum of 10 samples for each variant were used for each test.

### 2.3. Statistical Analyses

Analysis of variance (ANOVA) and *t*-test calculations were used to test (α = 0.05) for significant differences between factors and levels using the IBM SPSS statistic base (IBM, SPSS 20, Armonk, NY, USA). The means of shear strength of particular binders were compared in both native and polymerized wood, and homogenous and non-homogenous groups were indicated. Where applicable, the mean values of the investigated features and the standard deviation indicated as error bars are presented on plots as error bars.

## 3. Results

### 3.1. WPG Parameters

The WPG is the parameter that accounts for the weight gain of samples after polymerization with styrene. It is the primary parameter for evaluating polymerization [51]. The polymerization results of the samples are shown below.

Table 1 shows the average batch density before polymerization and the average WPG for samples after polymerization. In addition, the standard deviation for the averages was calculated in the tables. As presented, the average density of beech and alder wood before polymerization was 720 kg m^−3^ and 535 kg m^−3^, respectively. These values are similar to those obtained by other researchers [54]. In the case of alder, the values are slightly higher than Alma et al. [55]. The WPG parameter is 44.5% for beech and 87.8% for alder. The above polymerizations for both species settled at a higher level of WPG than in the study by Alma et al. [55].

### 3.2. Adhesive Dry Content

**Figure 1:**
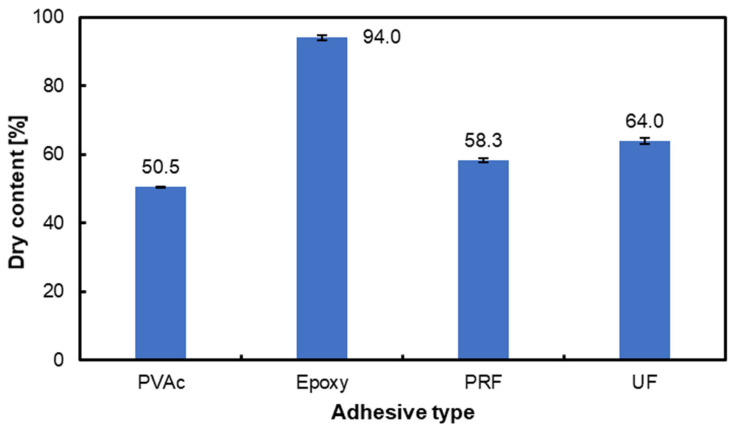
Dry matter content in adhesives intended for testing.

Figure 1 presents the dry content (%) of four adhesive types: PVAc, Epoxy, PRF, and UF. Among the adhesives, epoxy exhibits the highest dry content at 94.0%, followed by UF (64.0%), PRF (58.3%), and PVAc (50.5%). The data include error bars representing the standard deviation, highlighting the variability in measurements for each adhesive type.

The dry mass content of PVAc glue is crucial as it affects its functionality and adhesive features [56]. Furthermore, it is vital to comprehend the dry matter content to optimize the formulation and guarantee the intended adhesive characteristics [57]. The adhesive properties of epoxy glue are influenced by its dry mass content, with a decrease in physical properties observed as the water content rises [58]. Moreover, the moisture diffusivity in the epoxy adhesive is strongly impacted by salt concentration, emphasizing the role of dry mass content in determining the material’s behavior under varying environmental conditions [59]. PRF adhesives can have a range of solid contents. A PRF glue with a solid percentage of 14.7% is mentioned in one study [60]. A different investigation into wood treated with phenol-formaldehyde (PF) reported solid levels of 10% and 25% [61]. A lignin-modified phenol-formaldehyde resin adhesive (LPF) also included 51.2% solids [62]. PRF adhesives can also differ in their resorcinol concentration. One study, for example, looked at PRF adhesives that included 15% and 25% resorcinol [63,64]. UF glue usually has a solid percentage of between 45% and 63% [65]. The UF adhesive’s viscosity, bonding strength, and formaldehyde emission are all influenced by its solid concentration. For example, enhanced bonding strength and increased viscosity are often associated with a more significant solid contents [66]. 

Summarizing the dry matter content based on a comparison of literature data, it is evident that the information is not recent, with some sources dating back over a decade. Technological advancements and an improved understanding of adhesive formulations have likely influenced their compositions, which may impact the reported dry matter content during this time.

### 3.3. Shear Strenght

**Figure 2:**
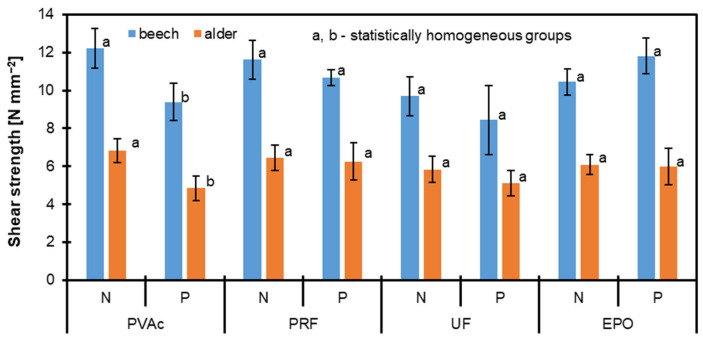
Shear strength of beech and alder wood bonding lines with PVAc, PRF, UF, and EPO adhesives under unpolymerized/native (N) and polymerized (P) conditions.

**Figure 3:**
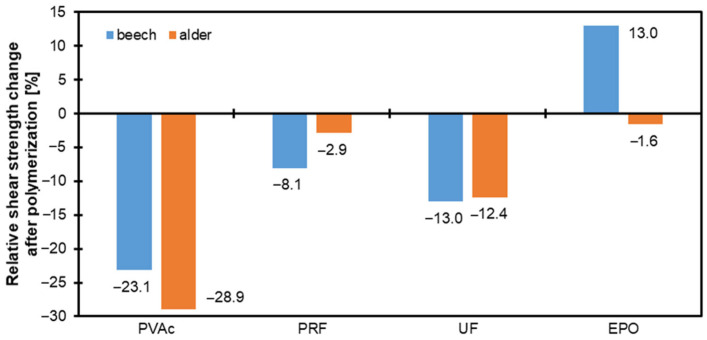
Relative shear strength of beech and alder wood bonded with PVAc, PRF, UF, and EPO adhesives after polymerization.

**Figure 4:**
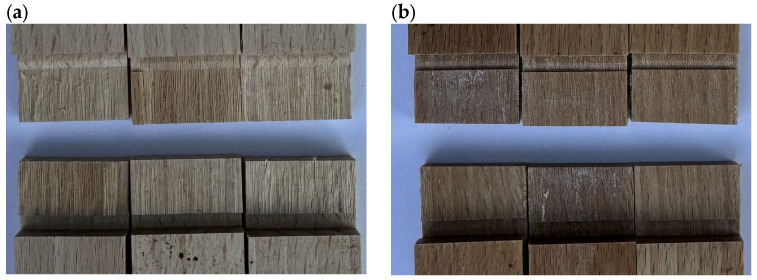

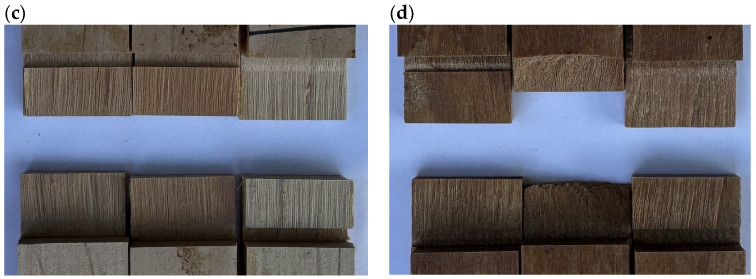
The UF-bonded samples after shear strength test: (**a**) beech, native; (**b**) beech, polymerized; (**c**) alder, native; and (**d**) alder, polymerized.

Figure 2 and Figure 3 illustrate the shear strength of two wood species, beech and alder, after treatment with four types of adhesives: PVAc, PRF, UF, and EPO. The samples are categorized into unpolymerized/native (N) and polymerized (P) types, and polymerization was achieved using styrene in the latter. Beech (blue bars) consistently exhibited higher shear strength than alder (orange bars) across all adhesives and treatment conditions. The pictures of the samples bonded with UF are presented in Figure 4. Since all the reference samples (native, non-modified by styrene polymerization) had a fully damaged wood structure, some brief information about the destruction of the polymerized samples during the shear strength test is provided in Table 2. Across both wood species and the data provided in the mentioned table, EPO and PRF consistently outperformed the other adhesives, with EPO excelling in beech and PRF in alder. This suggests that while both adhesives are highly effective, their performance may vary depending on the specific substrate properties of the wood species. PVAc demonstrates a medium level of bonding efficiency for both woods, making it a potential choice for applications where moderate adhesion is sufficient. On the other hand, UF consistently underperforms across both wood types, indicating its limitations as a wood adhesive for achieving high in-wood destruction ratios. These findings emphasize the importance of selecting adhesives based on compatibility with the wood species to optimize bond strength and durability in wood-based applications.

Among the tested adhesives, PVAc and Epoxy glue delivered the highest shear strength for both wood species, with beech achieving peak performance. PRF and UF resulted in comparatively lower shear strength values, particularly for alder, which displayed the weakest adhesion. Polymerization generally enhanced shear strength for both wood types, indicating improved adhesive bonding. However, the magnitude of improvement varied depending on the adhesive and wood species. The error bars indicate the measurements’ standard deviation, reflecting the shear strength variability within each treatment group.

Wood treated with styrene has better dimensional stability and absorbs less water, which prevents swelling [20,41,67]. In this study, this information is essential as it indicates that the penetration of glue in modified wood could be hindered, potentially affecting its adhesion. Additionally, increased hardness may be associated with greater brittleness, which might be undesirable for this test. According to scanning electron microscopy (SEM), styrene polymerization gives the wood structure a smoother texture and improved adherence [43,68]. Completely smooth surfaces are more challenging for glue; therefore, surface preparation often involves matting to enhance adhesion. This step was also carried out while preparing all samples for gluing. It was discovered that the density of the specimens greatly affected the shear strength of heat-treated solid wood bonded with polyvinyl acetate (PVA) adhesive augmented by nanowollastonite (NW), with both heat treatment and the presence of NW affecting the shear bond strength [69].

Regarding the epoxy binder that provided the best results when bonding styrene-impregnated wood, it should be pointed out that styrene-impregnated wood has a non-polar and hydrophobic surface due to the polymerization of styrene, which reduces surface energy and complicates adhesion. Epoxy resin can form strong bonds with such surfaces because it has a reactive epoxy group capable of forming covalent bonds with functional groups on the impregnated wood surface or styrene residues. Epoxy resin has an inherently high cohesive strength due to its crosslinked network structure after curing. This makes the bond more robust and durable, even when applied to challenging surfaces such as styrene-treated wood [70]. Its adhesive properties are less dependent on the polarity of the substrate compared to PVAc, UF, or PRF.

### 3.4. Density Profiles

**Figure 5:**
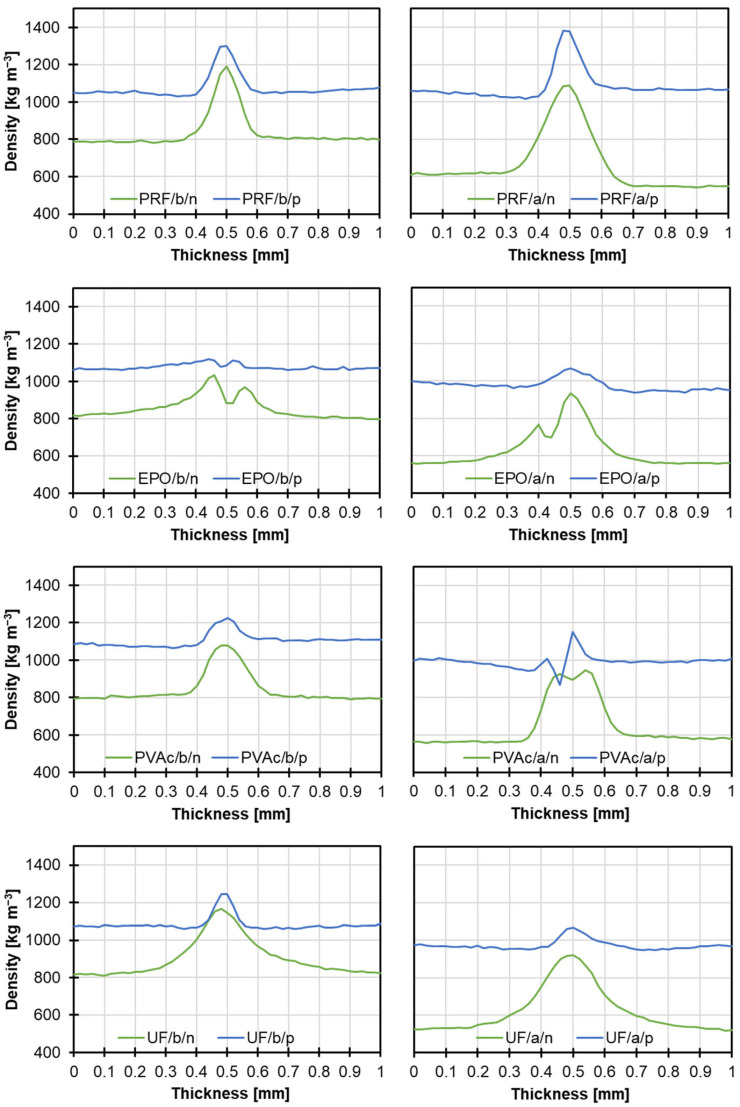
Density profiles of various wood bonding lines before and after modification (b—beech; a—alder; n—native; p—polymerized).

Figure 5 presents the density profiles of bonded beech and alder wood samples before and after polymerization. Each graph shows that the polymerized samples increased their density by approximately 200 kg m^−3^ to 400 kg m^−3^, depending on the wood species. Thickened wood provides a more uniform and consistent surface for glue application. This can result in a smoother and more uniform glue line, as evidenced by narrower and sharper profile peaks. Epoxy glue, due to its high dry matter content, exhibited the least penetration into polymerized wood, resulting in an almost flat glue line on the density profile.

Interestingly, despite this limited penetration, it was the only adhesive with the highest shear strength. Epoxy resins’ high adherence to various substrates is mainly due to their polar nature. Better bonding results from this polarity’s enhancement of the adhesive–substrate contact [70]. The curing agent impacts the epoxy’s strength, hardness, durability, and adhesive strength [71]. Epoxy adhesives’ strength is closely correlated with the substrates’ surface area and cleanliness; a larger surface area facilitates the creation of more robust connections between materials [72]. Although cured epoxy resins’ high cross-linking density makes them brittle, it also improves their mechanical strength and adhesive qualities [73,74]. The explanation for UF glue and others that performed poorly in the shear strength tests could lie in a different wood polymerization process, such as acetylated wood. Acetylated wood has a hydrophobic surface, which limits the adhesive’s wettability and the formation of chemical bonds between the resin and the wood polymers [75].

Based on the studies conducted, the following suggestions arise for further investigation. Future studies should concentrate on purifying the monomer and initiator before polymerization to avoid potential inhibitors that may interfere with the reaction. Tests using purified reagents would yield more accurate results regarding polymerization efficiency and contribute to a more controlled process. Additionally, exploring different initiators and monomers could increase process efficiency and control while also influencing the mechanical properties of the wood. These efforts could significantly enhance the potential of styrene-modified timber across various industries.

## 4. Conclusions

Based on the conducted research, the following conclusions were reached:

- The epoxy adhesive exhibits the highest dry content among tested adhesives, correlating with its superior shear strength and limited penetration into polymerized wood. This suggests that a higher dry mass content enhances adhesive performance, especially in dense, modified wood structures. In contrast, adhesives with lower dry content, such as UF, do not perform as well due to reduced bonding and penetration capabilities.
- Polymerization using styrene improves the shear strength of the epoxy bonding line for beech wood. Beech consistently outperforms alder in shear strength due to its denser structure. However, polymerization and surface modifications, such as smoothing by styrene, can reduce adhesive penetration, necessitating additional surface preparation to optimize bonding.
- Advances in adhesive formulation and wood treatment technologies influence adhesive performance. Epoxy adhesive demonstrates exceptional shear strength despite limited penetration, highlighting the importance of adhesive–substrate interactions. Variability in performance across adhesives and wood types emphasizes the need for tailored approaches, considering factors like adhesive composition, wood density, and surface preparation.

## Figures and Tables

**Table 1:** The density before polymerization and WPG for the beech and alder wood.

Wood Species	Density Before Modification [kg m^−3^]		WPG [%]	
	Average	Standard Deviation	Average	Standard Deviation
Beech	720	7	44.5	3.5
Alder	535	16	87.8	5.3

**Table 2:** The brief information about the destruction of the polymerized samples during the shear strength test.

Wood Species	In-Wood Destruction Area Ratio [%]			
	PVAc	PRF	UF	EPO
Beech	70	80	40	100
Alder	60	100	50	90

## Data Availability

The data presented in this study are openly available in RepOD https://doi.org/10.18150/IK9DII (created and accessed on 23 November 2024).
